# Lipidomic Analyses, Breast- and Formula-Feeding, and Growth in Infants

**DOI:** 10.1016/j.jpeds.2014.10.021

**Published:** 2015-02

**Authors:** Philippa Prentice, Albert Koulman, Lee Matthews, Carlo L. Acerini, Ken K. Ong, David B. Dunger

**Affiliations:** 1 Department of Pediatrics, University of Cambridge Metabolic Research Laboratories Wellcome Trust-Medical Research Council Institute of Metabolic Science, National Institute of Human Research Cambridge Comprehensive Biomedical Research Center, Cambridge, United Kingdom; 2 Medical Research Council Human Nutrition Research, Cambridge, United Kingdom

**Keywords:** CBGS, Cambridge Baby Growth Study, CE, Cholesterol ester, DBS, Dried blood spot, LC-PUFA, Long chain polyunsaturated fatty acid, PC, Phosphatidylcholine, PC-O, 1-alkyl,2-acylglycerophosphocholine, PC-P, 1-(alkenyl),2-acylglycerophosphocholin, PLS-DA, Partial least squares-discriminant analysis, SM, Sphingomyelin, TG, Triglyceride

## Abstract

**Objective:**

To evaluate lipidomic differences between breast- and formula-fed infants.

**Study design:**

We utilized high-resolution mass-spectrometry methods to analyze 3.2 mm dried blood spot samples collected at ages 3 months (n = 241) and 12 months (n = 144) from a representative birth cohort study. Lipidomic profiles were compared between infants exclusively breast-fed, formula-fed, or mixed-fed, and related to 12-month infancy weight. Data analysis included supervised multivariate statistics (partial least squares discriminant analysis), and univariate analysis with correction for multiple testing.

**Results:**

Distinct differences in 3-month lipidomic profiles were observed between exclusively breast-fed and formula-fed infants; mixed-fed infants showed intermediate profiles. Principle lipidomic characteristics of breast-fed infants were lower total phosphatidylcholines (PCs), with specifically lower short chain unsaturated PC but higher long chain polyunsaturated PC; higher cholesterol esters; and variable differences in sphingomyelins. At 12 months, lipidomic profiles were markedly different to those at 3 months, and differences between the earlier breast/formula/mixed-feeding groups were no longer evident. However, several specific lipid species, associated with breast-feeding at 3 months, also correlated with differences in 3- to 12-month weight.

**Conclusions:**

State-of-the-art dried blood spot sample lipidomic profiling demonstrated striking differences between breast-fed and formula-fed infants. Although these changes diminished with age, breast-fed lipidomic profiles at 3 months were associated with infancy weight and could potentially represent biomarkers of infant nutrition.

Links between early life exposures and later health outcomes may in part be due to nutritional programming in infancy. This hypothesis is supported by observed long-term benefits associated with breast-feeding, such as better cognitive development in childhood, and lower risks of obesity and high blood pressure in later life.^1^ Effects of early nutritional interventions in infancy, using nutrient-enriched milk formulas, include increased later risk of metabolic disease.^2^ However, the underlying mechanisms are unknown and are difficult to study.

Previous work has shown that the biochemical phenotype of infants differs according to feeding practice (breast- vs formula-feeding). Higher total cholesterol levels,^3^, ^4^, ^5^, ^6^, ^7^ higher low density lipoproteins,^5^ and lower high density lipoproteins^7^ have been reported in breast-fed infants, and may lead to lower cholesterol levels in adulthood.^5^, ^8^, ^9^ However, more detailed lipidomic profiling in breast- vs formula-fed infants has not yet been performed. We, therefore, utilized technological developments in lipidomics for metabolic phenotyping^10^ to obtain a detailed lipidomic profile from dried blood spot (DBS) samples in infants recruited into the Cambridge Baby Growth Study (CBGS). Our aim was to investigate the relationships between infancy feeding and age on specific lipids across multiple lipid classes.

## Methods

The CBGS is a prospective observational birth cohort, focusing on the antenatal and early postnatal determinants of infancy growth. Mothers were recruited during early pregnancy from a single antenatal center in Cambridge between 2001 and 2009.^11^ Their infants were seen at birth by trained research nurses and then followed-up at 3 and 12 months of age, with weight measurements. DBS samples were collected when parents consented, using capillary heel prick sampling, dropping blood spots onto untreated filter paper cards (Ahlstrom 226; ID Biological Systems, Greenville, South Carolina). Infancy feeding (exclusive breast-, mixed-, or exclusive formula-feeding) was assessed by questionnaire at age 3 months. All children were on a full mixed diet by 12 months of age. The study was approved by the Cambridge local research ethics committee, and all mothers gave informed written consent.

DBS samples on filter paper cards were air dried at ambient temperature overnight, before being stored in zip-loc storage bags at −20°C until analysis, when a single 3.2 mm spot was punched from the larger DBS samples. Individuals with sufficient DBS samples for multiple analyses were selected for this nested study.

We recently reported that large-scale lipidomic profiling platforms established for use on plasma samples^10^ can be successfully adapted to DBS samples.^12^ In brief, a 3.2 mm DBS was extracted in methanol in a well of a glass-coated 2.4 mL deep well plate (Plate+TM; Esslab, Hadleigh, United Kingdom), and lipids were partitioned into methyl tertiary butyl ether. After centrifugation, the organic layer was concentrated and used for lipid analysis. Samples were infused into a Thermo Exactive benchtop orbitrap (Hemel; Hampstead, United Kingdom), using an Advion Triversa Nanomate (Ithaca, New York) and data acquired in both positive (1.2 kV voltage) and negative modes (−1.5 kV). All experiments were run with blank controls and 2 different quality control samples. In total, 94 lipid species could be detected using this method.

### Statistical Analyses

Lipidomic profiles were compared between infants exclusively breast-fed, mixed-fed, and formula-fed, between age 3 vs 12 months, and also related to infancy weight at age 12 months. The obtained lipid signals were semiquantitative, with the signal intensity of each lipid expressed relative to the total lipid signal intensity, for each individual, per thousand (‰). Raw high-resolution mass-spectrometry data were processed using XCMS (www.bioconductor.org) and Peakpicker v 2.0 (an in-house R script). All lipid species where >30% of cases had a value of zero were excluded from further analyses; consequently, 78 lipids were analyzed at 3 months, and 90 lipids were compared between 3- and 12-month samples. Multivariate analysis allowed analysis of multiple variables (all lipid species) together, to avoid loss of information and to identify underlying trends. These data were analyzed in SIMCA-P (v. 13; Umetrics AB, Umeå, Sweden). Data were normalized using log transformation and unit variance scaled. Principal component analysis was used first to observe overall patterns and detect outliers. Partial least squares-discriminant analysis (PLS-DA) plots were then constructed, using Q^2^ values for cross validation. The PLS-DA algorithm was chosen as this maximizes separation between lipid variables for each categorical outcome (infancy feeding/age), enabling clear determination of variables contributing to any separation. Receiver operating characteristic curves were plotted to assess the predictive ability of obtained models (http://www.roccet.ca/).^13^ Univariate analysis, using Mann-Whitney U for exclusively breast-fed vs formula-fed infants, and Wilcoxon signed ranks tests for matched age 3 vs 12 month samples, was performed in SPSS v 19 (SPSS Inc, Chicago, Illinois). A stringent Bonferroni corrected *P* value (<.0006) was used to identify significant associations with individual lipids. This was calculated by dividing the significance threshold of .05 by the number of variables, in this case the lipids analyzed (78 at 3 months; 90 comparing 3 and 12 months). Spearman correlations were used for correlations between DBS lipids and infancy weight.

## Results

Two hundred forty-one infants (110 male) had DBS lipidomic profiles measured at age 3 months (mean ± SD: 97 ± 9 days of age); 141 infants (77 male) at 12 months (373 ± 12 days), with 45 paired samples from the same infants at both time points. The cohort characteristics are summarized in Table I, and the sample was representative of the total CBGS (N = 1340 at 3 months), with mean ± SD birth weight 3.49 ± 0.55 kg, at 39.8 ± 1.6 weeks gestation, 3-month weight 6.15 ± 0.83 kg, and 12-month weight 9.97 ± 1.19 kg.

**Table I:** Demographics of infancy subgroups at 3 and 12 months of age

	All 3 months (N = 241), mean ± SD	3-month breast-fed (N = 81), mean ± SD	3-month formula-fed (N = 84), mean ± SD
Sex (% male)	46%	42%	46%
Gestational age (wk)	39.9 ± 1.5	40.2 ± 1.3	39.8 ± 1.5
Birth weight (kg)	3.50 ± 0.52	3.54 ± 0.43	3.50 ± 0.57
3 m weight (kg)	6.13 ± 0.80	5.96 ± 0.66	6.38 ± 0.76∗
3 m weight SDS	−0.07 ± 1.05	−0.19 ± 0.88	0.25 ± 0.96∗

	All 12 mo (N = 141), mean ± SD	12 mo initially breast-fed (N = 38), mean ± SD	12 mo initially formula-fed (N = 50), mean ± SD
Sex (% male)	55%	47%	56%
Gestational age (wk)	40.0 ± 1.4	40.1 ± 1.2	39.9 ± 1.4
Birth weight (kg)	3.47 ± 0.52	3.58 ± 0.43	3.43 ± 0.62
12 m weight (kg)	10.03 ± 1.31	9.72 ± 1.26	10.32 ± 1.41∗
12 m weight SDS	0.07 ± 1.21	−0.19 ± 1.20	0.34 ± 1.24∗

∗ *P* < .05 between breast-fed and formula-fed infants.

Infants transported the majority of long chain polyunsaturated fatty acids (LC-PUFAs) as phospholipids, rather than as cholesterol esters (CEs) or triglycerides (TGs). Overall in these infant samples, phosphatidylcholines (PCs) contributed 35% of the total lipid signal; TGs in contrast only contributed to approximately 10% of the total lipid signal. DBS samples had been stored for 2.1-9.5 years at −20°C. Duration of storage was significantly positively correlated with only 3 lipid species: lyso PC(18:0), PC(32:0), and PC(34:3); none were inversely correlated. There was no significant difference between the lipidomic profiles of male and female infants, at either 3 or 12 months of age.

**Figure 1:**
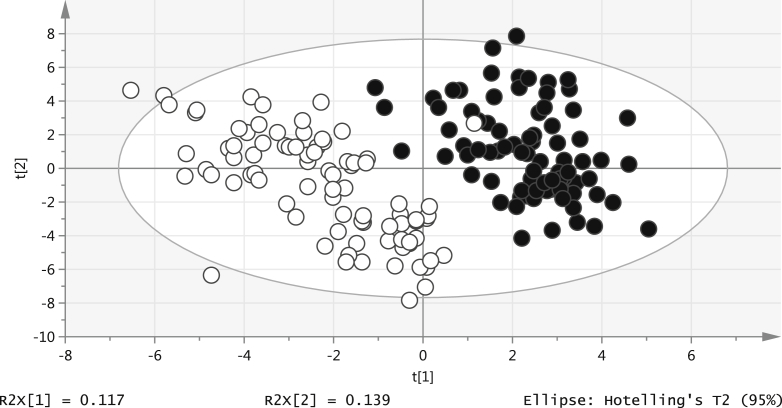
PLS-DA plot: 3-month lipids and nutrition (*black*: breast-fed; *white*: formula-fed).

**Figure 2:**
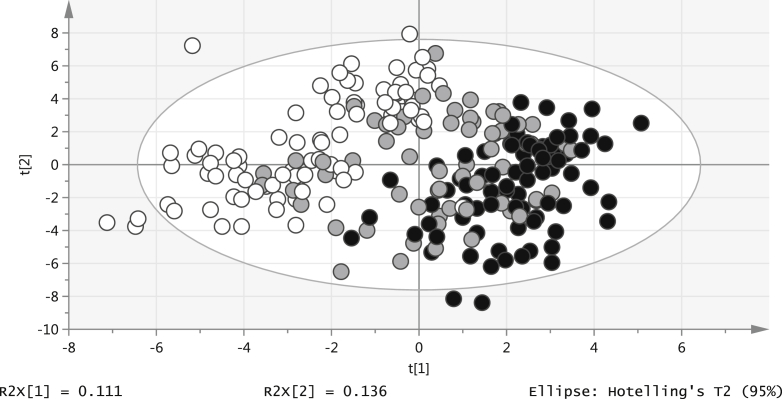
PLS-DA plot: 3-month lipids and nutrition (*black*: breast-fed; *white*: formula-fed; *gray*: mixed-fed).

Of the 241 infants at 3 months of age, 81 were exclusively breast-fed, 66 mixed-fed, and 84 formula-fed (4 infants receiving solid food and 6 infants with unknown feeding type were excluded from further analysis). Table I shows the marked anthropometric differences between exclusively breast- and formula-fed infants, with higher weight in formula-fed infants. In multivariate analysis, there was a clear difference between the 3-month lipidomic profiles of exclusively breast-fed vs exclusively formula-fed infants (Q^2^ = 0.816; Figure 1), with mixed-fed infants showing an intermediate profile (Q^2^ = 0.337; Figure 2; available at www.jpeds.com).

Using univariate analysis, 30 individual lipid species showed significant differences between exclusively breast-fed and solely formula-fed infants (below the multicomparison corrected threshold: *P* < .0006) (Table II; available at www.jpeds.com). Considering total levels of 3 main lipid classes, the total PC level was lower in breast-fed compared with formula-fed infants: mean values 350‰ vs 385‰ (*P* < .0005), but there were no differences in total sphingomyelins (SMs) or TGs.

**Figure 3:**
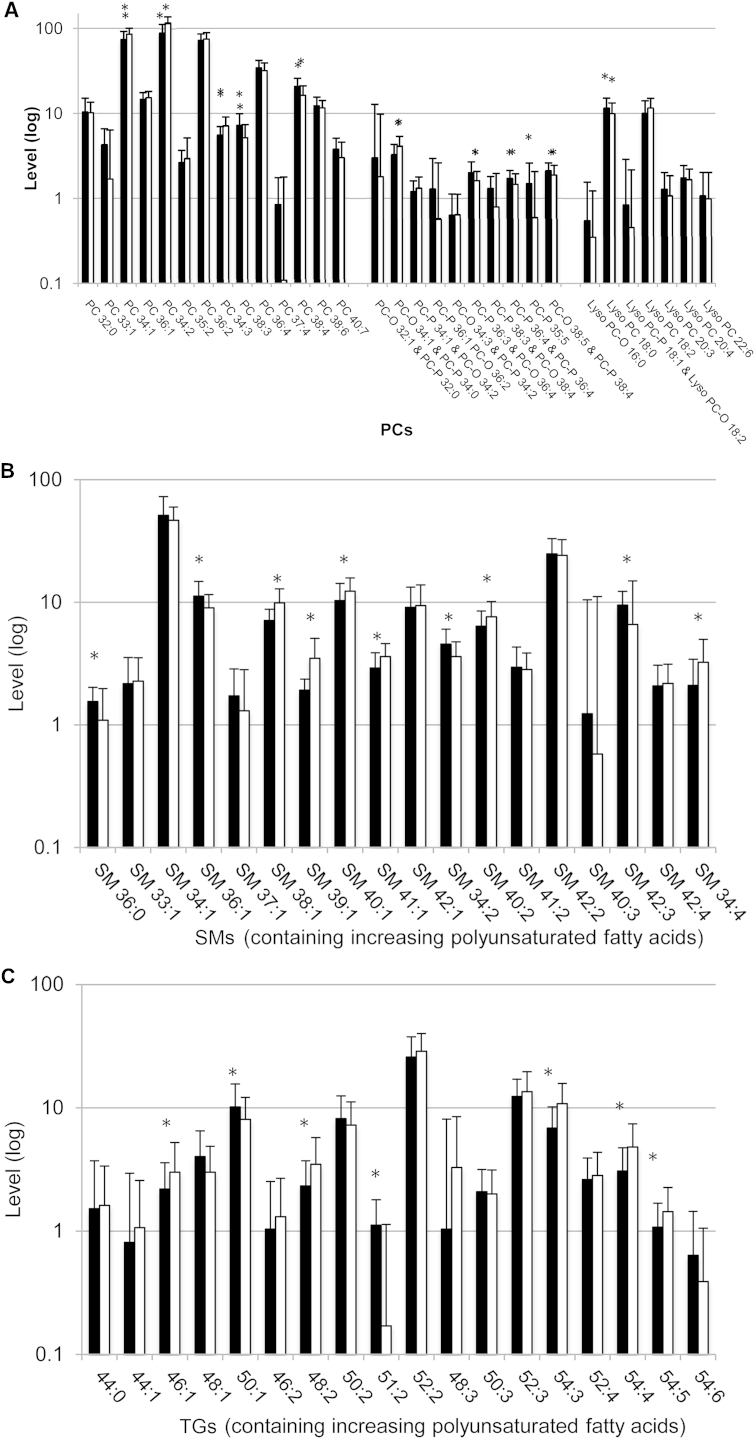
Levels of **A,** PCs; **B,** SMs; and **C,** TGs (median ± IQR) at 3 months of age in breast- and formula-fed infants. ^∗^*P* < .0006 (*black*: breast-fed; *white*: formula-fed).

Looking in more detail at these lipidomic profiles (Figure 3), breast-fed infants had lower short chain unsaturated PCs but higher longer chain polyunsaturated fatty acid containing PCs. In addition, there were multiple individual differences within the TG and SM lipid classes. Seven TGs were significantly different between exclusively breast-fed and formula-fed infants, with lower levels of polyunsaturated fatty acid containing long chain TGs in the breast-fed infants. There were several higher shorter chain SMs in the breast-fed infants, but lower levels of many of the longer chain SMs. Breast-fed infants had higher CE(16:0) and CE(20:4) levels.

Levels of 5 specific DBS lipids were sufficient to distinguish between infant feeding patterns (exclusive breast-vs formula-feeding) at an area under the receiver operating curve = 0.95 (95% CI: 0.879-0.997); these were PC(34:2) (lower in breast-fed), SM(34:4) (lower in breast-fed) SM(36:1) (higher in breast-fed), TG(50:1) (higher in breast-fed), and TG(51:2) (higher in breast-fed).

**Figure 4:**
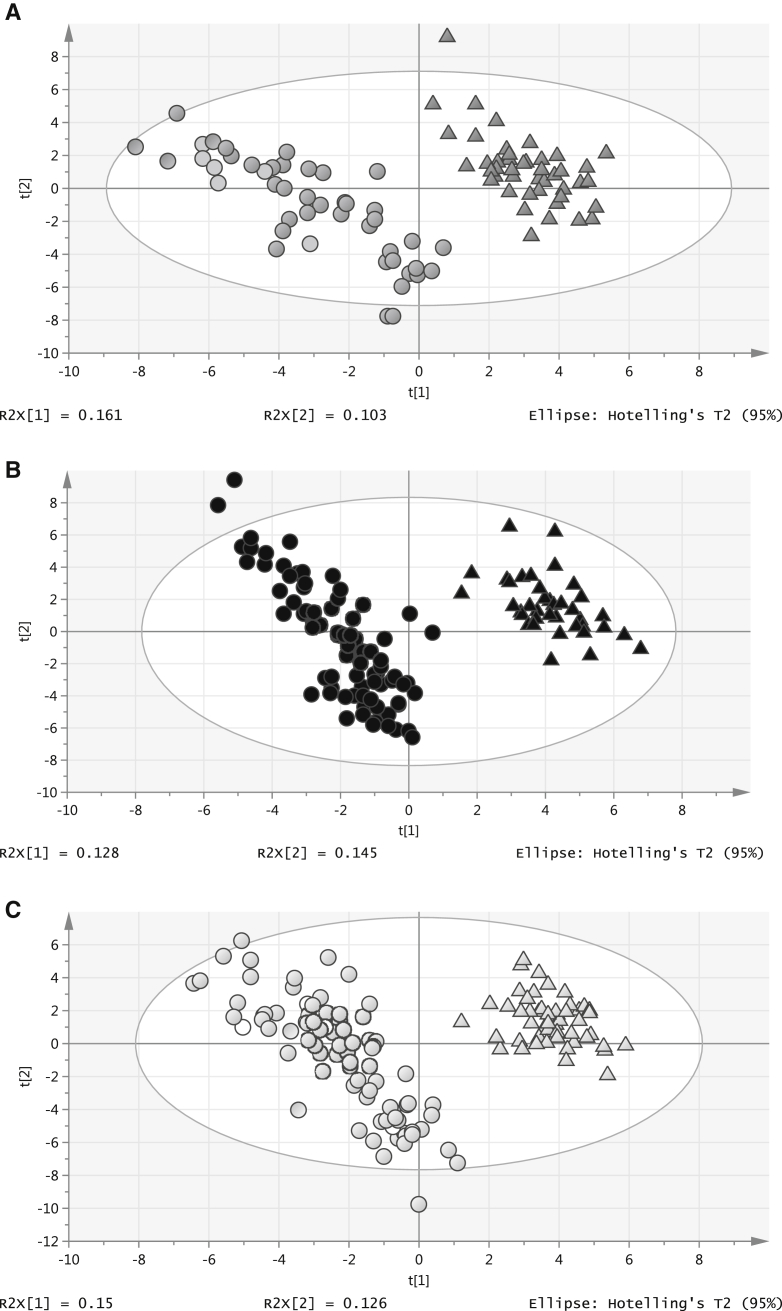
**A,** PLS-DA plot: DBS lipids at 3 and 12 months (45 paired samples, *circles*: 3 months; *triangles*: 12 months). **B,** PLS-DA plot: 3- and 12-month lipids in breast-fed infants (*circles*: 3 months; *triangles*: 12 months). **C,** PLS-DA plot: 3- and 12-month lipids in formula-fed infants (*circles*: 3 months; *triangles*: 12 months).

There were large differences between the lipidomic profiles at 12 months compared with 3 months of age. In the 45 paired DBS samples at both 3 and 12 months of age (Q^2^ = 0.916), 25 lipids showed significant age differences (*P* < .0006 with Wilcoxon signed ranks tests) (Figure 4, B and Table III; available at www.jpeds.com). These differences were also seen among the larger number of unpaired samples at 3 and 12 months, and additionally, when infants were separated into those exclusively breast-fed for at least 3 months, and those exclusively formula-fed, as shown in the multivariate models (Q^2^ = 0.922; Figure 4, B and C).

**Figure 5:**
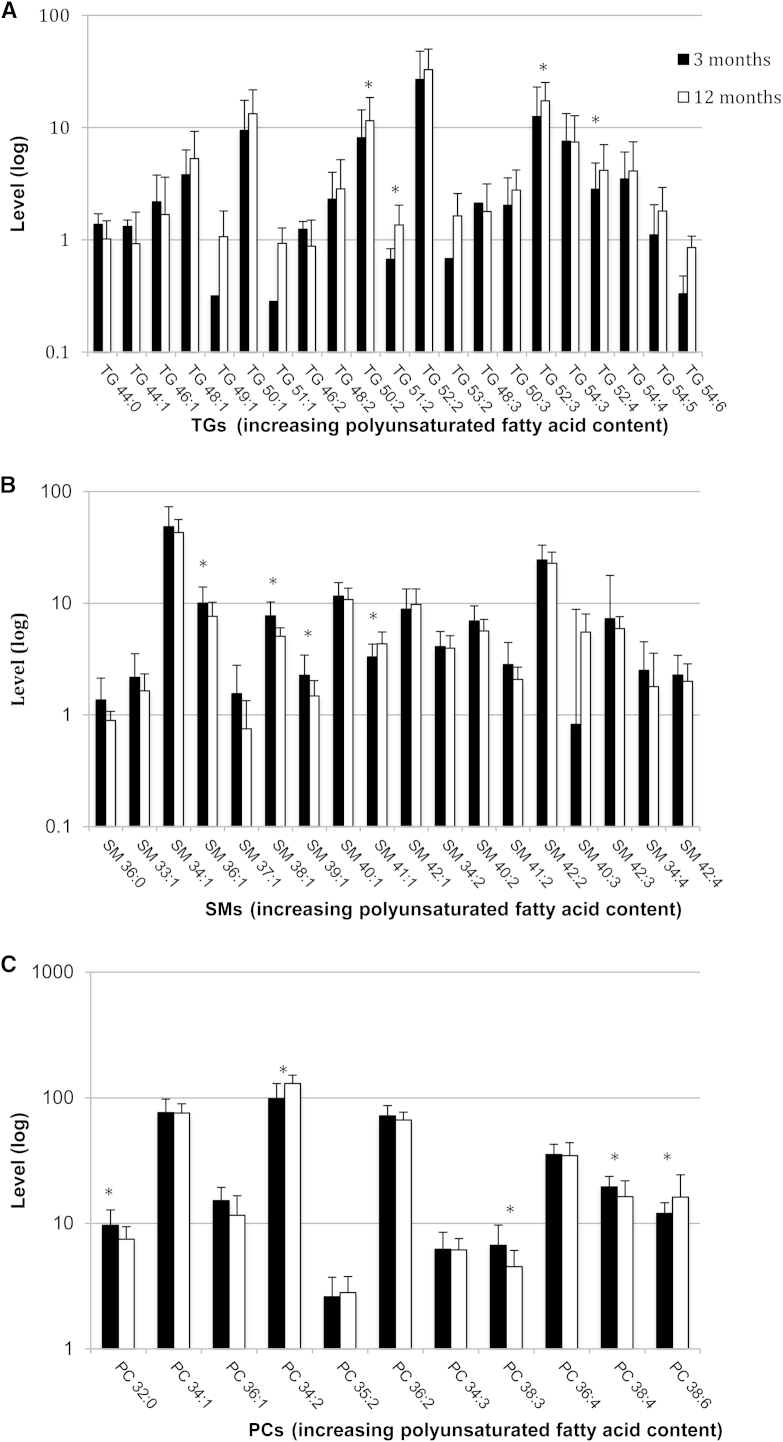
Levels of lipid classes in paired samples at 3 and 12 months of age**. A,** TGs; **B,** SMs; and **C,** PCs (median ± IQR). ^∗^*P* < .0005.

Overall, there was a trend towards higher total PCs at age 12 months (377 vs 360, *P* = .06), but with variable differences in specific PC species (Figure 5; available at www.jpeds.com). PC class differences at 12 months, compared with 3 months, included higher PC(34:2) containing palmitate, {with an increased ratio of palmitate [PC(34:2)] to stearate [PC(36:2)], *P* < .0005}, higher omega-3 containing PC 38:6 and lower omega-6 containing PC 38:4. Total CE (55 vs 45, *P* < .0005) and specifically CE(18:2) was higher at 12 months, as were TGs, (127 vs 108, *P* = .04), particularly long chain TGs (the majority polyunsaturated). Total SMs was lower at 12 months (144 vs 164, *P* = .02).

**Figure 6:**
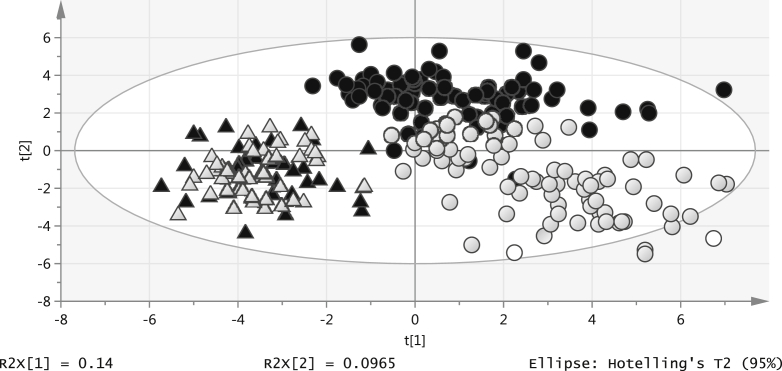
PLS-DA plot: 3- and 12-month DBS lipids (*circles*: 3 months; *triangles*: 12 months; *black*: breast-fed; *white*: formula-fed).

Two DBS lipid signals were enough to predict age: lyso PC(20:3) (higher at 12 months) and 1-alkyl,2-acylglycerophosphocholine (PC-O) (34:1) [which cannot be distinguished from 1-(alkenyl),2-acylglycerophosphocholine (PC-P) (34:0)^12^ (lower at 12 months)], creating a robust PLS-DA model with separation in 1 component (Q^2^ = 0.791). Of note, the separation seen clearly between infant feeding groups at 3 months was no longer apparent at 12 months of age (Q^2^ = 0.549; Figure 6).

Additional analyses of 3-month profiles investigated correlations between those lipid species differing between breast- and formula-fed infants, and 3-month, and 12-month weight SDS, as well as change in 3- to 12- month weight SDS. Infancy weight was positively associated with PC(34:1) (r_s_ 0.2, *P* < .005 at 3 months, r_s_ 0.2, *P* = .003 at 12 months) and PC-O(34:1) (r_s_ 0.2, *P* = .003 at 3 months, r_s_ 0.2, *P* = .009 at 12 months), and inversely associated with PC(38:4) (r_s_ −0.2, *P* < .0005 at 3 months, r_s_ −0.2, *P* = .002 at 12 months) (Table IV; available at www.jpeds.com), with no significant association seen for the other lipid species that differed between breast- and formula-fed infants. Although these associations were no longer significantly related to 3- to 12-month weight gain, 2 additional lipid species were inversely associated with weight gain: SM(34:2) (r_s_ −0.2, *P* = .005) and PC-O(36:4) [which cannot be distinguished from PC-P(36:3)^12^] (r_s_ −0.2, *P* = .004) (Table IV; available at www.jpeds.com).

## Discussion

A wide variety of lipid classes contributed to the total lipidomic signal. We also demonstrate that, unlike adults, infants transport LC-PUFAs mainly as phospholipids [eg, PC(38:4), PC(38:6)], rather than as CEs and TGs.^14^ At 3 months, PCs contributed to 35% of the total lipid signal, with the majority being medium and long chain-fatty acids. However, it is also important to note that our study investigated DBS samples, whereas most data in adults include only serum or plasma circulating lipids. We observed no sex differences, again differing with findings in later life.^15^, ^16^

We found clear differences in lipidomic profiles by early feeding pattern, with differences in specific lipids across all main lipid classes between exclusively breast- and formula-fed infants. Notably, formula and breast milk are not dissimilar in their lipid composition, consisting primarily of TGs,^17^, ^18^ (with a larger diversity of fatty acids in the TGs of breast milk^19^). Therefore, the infancy lipidomic profiles do appear to not only reflect the lipid composition of the milk intake, but also there may be additional effects of infant nutrition on early fat metabolism.

The fatty acid composition of plasma and erythrocyte membranes lipids has been previously measured in children, using gas chromatography, showing some fatty acid variation with age, for example increasing linoleic acid and docosahexaenoic acid from infancy to young adulthood.^20^ However, we show that high-resolution mass-spectrometry provides a more comprehensive overview of lipid metabolism. We demonstrated very specific variation, including differences in the level of saturation and carbon length for each lipid class, and adding detail to overall total lipid class and fatty acid levels. For example breast-fed, compared with formula-fed infants had lower short chain unsaturated PC but higher LC-PUFA containing PC. This extends previous but limited knowledge, mainly focused on cholesterol and lipoprotein levels, into breast and formula milk differences on early physiology and metabolism. It is well documented that breast-fed infants have higher total cholesterol^3^, ^4^, ^5^, ^6^, ^7^ and low-density lipoprotein levels^5^ but lower high-density lipoprotein.^7^ We found higher CEs CE(16:0) and CE(20:4) in breast-fed infants. Higher omega 3- and 6-fatty acids in breast-fed infants have also been reported.^21^ We found no difference in the omega-3 containing PC 38:6, but showed higher omega-6 containing PC 38:4 levels in breast-fed infants.

What constitutes a favorable or “healthy” infancy lipidomic profile has yet to be established. However, lipid differences between exclusively breast- and formula-fed infants may contribute to some of the benefits of breast milk intake, including lower risk of infection, higher childhood cognitive development, and lower obesity risk.^1^ For example, higher proinflammatory lyso-PC levels in breast-fed infants could potentially contribute to lower morbidity from infectious disease.^22^ PC and SMs are both major contributors to cell membrane structure. SMs particularly are essential for specialized membrane formation in brain cells, signaling pathways and the immune response,^23^ and SM intake has previously been associated with improved neurodevelopment in preterm infants.^24^

Previously reported changes in lipidomic profile with age include CEs, and omega-3 and -6 PUFAs.^25^, ^26^ We build on this, showing changes in these lipid levels and other striking differences across the whole lipidomic profile, with generally lower SMs and higher TGs at 12 vs 3 months of age, again with specific changes within all lipid classes. Our previous pilot work showed large PC differences, with increased PC(34:2) containing palmitate at 12 months,^12^ and here we confirm an increased palmitate:stearate at 12 months. We also demonstrate that more PUFAs are transported as TGs, with lower levels of PUFA-containing PC, than at 3 months of age.

In agreement with previous small studies, which reported that differences in cholesterol levels between breast- and formula-fed infants attenuated after cessation of breast-feeding and introduction of complementary foods,^3^, ^27^ we demonstrated no effect of infant milk feeding at 3 months on lipidomic profile at 12 months. However, there may still be long-term programming effects. We found strong trends between 3 PCs at 3 months of age and infant weight at both 3 and 12 months; 3-month PC(34:1) and PC-O(34:1) were both positively related to infant weight and were also lower in exclusively breast-fed infants. The omega 6 containing PC(38:4) was inversely associated with weight and was also higher in exclusively breast-fed infants. These lipids were not significantly associated with 3- to 12-month weight gain and, therefore, are likely to reflect the maintenance of the difference in weight between breast-fed and formula-fed infants at 3-months. However, these PCs could still have potential value as biomarkers linking infant nutrition and growth. In addition, SM(34:2) and PC-O(36:4) were inversely related to 3- to 12-month weight gain.

Further study should assess whether lipids might predict ongoing weight gain and explore possible causal effects. Use of lipid profiling in nutritional intervention studies could also help to unravel these relationships and underlying mechanisms.

## Figures and Tables

**Table II:** Lipid differences at 3 months of age between exclusively breast-fed (N = 81) and exclusively formula-fed (N = 84) infants (median [IQR]) per thousand (‰)

	Exclusively breast-fed (N = 81)	Exclusively formula-fed (N = 84)	Mixed-fed (N = 66)
Total cholesterol	156 (42.0)	155 (29.6)	153 (46.0)
CEs			
CE 16:0∗	6.53 (3.04)	4.83 (2.6)	5.76 (2.08)
CE 18:1	14.3 (7.21)	16.3 (5.72)	15.9 (5.54)
CE 18:2	19.7 (7.94)	20.3 (6.76)	19.6 (8.28)
CE 20:4∗	1.8 (1.01)	1.28 (1.1)	1.55 (0.998)
Phospholipids			
PCs			
PC 32:0	10.4 (4.73)	10.2 (3.37)	9.73 (4.69)
PC 34:1∗	74.4 (17.7)	86.1 (14.6)	77.3 (17.0)
PC 34:2∗	88.6 (22.9)	116 (20.9)	101 (20.4)
PC 34:3∗	5.59 (1.42)	7.15 (1.94)	6.74 (1.90)
PC 35:2	2.65 (1.03)	2.94 (2.22)	2.45 (1.09)
PC 36:1	14.7 (2.88)	15.3 (2.86)	15.2 (3.82)
PC 36:2	72.5 (13.7)	75.2 (14.0)	72.6 (11.8)
PC 36:4	34.5 (7.56)	32.0 (7.28)	32.9 (6.72)
PC 37:4	0.847 (0.909)	0.110 (1.68)	0.630 (1.32)
PC 38:3∗	7.26 (2.66)	5.19 (2.20)	6.33 (2.51)
PC 38:4∗	20.9 (5.02)	16.4 (4.71)	19.0 (4.39)
PC 38:6	12.3 (3.27)	11.6 (2.58)	12.0 (4.09)
PC 40:7	3.79 (1.33)	3.03 (1.57)	3.58 (1.46)
Lyso-PC 18:0∗	11.6 (3.56)	9.98 (3.29)	10.7 (4.0)
Lyso-PC 18:2	10.1 (3.99)	11.6 (3.48)	11.1 (4.92)
Lyso-PC 20:3	1.28 (0.741)	1.07 (0.784)	1.17 (0.633)
Lyso-PC 20:4	1.74 (0.702)	1.66 (0.559)	1.72 (0.662)
Lyso-PC 22:6	1.07 (0.956)	0.984 (1.04)	1.20 (1.06)
Lyso-PC-O 16:0	0.545 (1.01)	0.353 (0.874)	0.645 (0.966)
Lyso-PC-P 18:1 and lyso-PC-O 18:2	0.836 (2.05)	0.451 (1.72)	0.560 (1.47)
PC-O 32:1 and PC-P 32:0	3.02 (9.76)	1.80 (8.02)	2.10 (9.01)
PC-O 34:1 and PC-P 34:0∗	3.28 (1.05)	4.17 (1.20)	3.44 (1.37)
PC-O 34:3 and PC-P 34:2	0.632 (0.495)	0.639 (0.484)	0.726 (0.458)
PC-O 38:5 and PC-P 38:4∗	2.12 (0.499)	1.89 (0.565)	1.94 (0.595)
PC-P 34:1 and PC-O 34:2	1.20 (0.412)	1.32 (0.467)	1.27 (0.528)
PC-P 35:5∗	1.49 (1.12)	0.594 (1.48)	1.32 (1.10)
PC-P 36:1 and PC-O 36:2	1.29 (1.67)	0.573 (2.05)	1.11 (1.55)
PC-P 36:3 and PC-O 36:4∗	2.02 (0.674)	1.62 (0.459)	1.78 (0.489)
PC-P 36:4∗	1.72 (0.415)	1.46 (0.492)	1.64 (0.536)
PC-P 38:3 and PC-O 38:4	1.31 (0.502)	0.792 (1.18)	1.19 (0.593)
PEs			
PE 36:3	1.32 (1.3)	1.11 (1.29)	1.41 (1.17)
PE 36:4	1.79 (2.39)	1.64 (1.77)	1.94 (2.47)
PE 40:2	2.24 (9.3)	1.51 (6.75)	2.73 (12.8)
Mixed PC and PE			
PC 33:1 and PE 36:1	4.29 (2.32)	1.69 (4.7)	4.27 (2.71)
PC 33:2 and PE 36:2	1.16 (0.754)	1.21 (0.729)	1.20 (0.635)
PC 35:1 and PE 38:1	4.97 (2.57)	2.51 (9.03)	5.26 (6.15)
PC 35:4 and PE 38:4	1.21 (0.888)	1.01 (0.87)	1.24 (0.725)
SMs			
SM 33:1	2.16 (1.39)	2.27 (1.27)	2.39 (1.44)
SM 34:1	51.1 (21.9)	46.6 (13.4)	48.9 (21.7)
SM 34:2∗	4.55 (1.49)	3.60 (1.16)	4.23 (1.34)
SM 34:4∗	2.10 (1.33)	3.23 (1.76)	2.63 (1.49)
SM 36:0∗	1.55 (0.476)	1.09 (0.894)	1.44 (0.71)
SM 36:1∗	11.2 (3.61)	8.99 (2.6)	10 (2.68)
SM 37:1	1.72 (1.14)	1.30 (1.52)	1.61 (1.31)
SM 38:1∗	7.09 (1.71)	9.88 (3.01)	7.94 (2.31)
SM 39:1∗	1.92 (0.446)	3.48 (1.62)	2.42 (0.842)
SM 40:1∗	10.3 (3.97)	12.3 (3.52)	11.2 (3.09)
SM 40:2∗	6.38 (2.13)	7.61 (2.57)	6.71 (2.51)
SM 40:3	1.23 (9.27)	0.577 (10.6)	1.04 (11.7)
SM 41:1∗	2.91 (0.98)	3.61 (1.01)	3.21 (0.663)
SM 41:2	2.95 (1.37)	2.82 (1.04)	2.82 (1.87)
SM 42:1	9.08 (4.26)	9.37 (4.51)	9.68 (4.02)
SM 42:2	24.8 (8.38)	24.1 (8.47)	24.5 (9.19)
SM 42:3∗	9.49 (2.79)	6.57 (8.43)	7.60 (9.56)
SM 42:4	2.08 (1.00)	2.18 (0.95)	2.11 (0.725)
TGs			
TG 44:0	1.52 (2.21)	1.62 (1.76)	1.91 (2.16)
TG 44:1	0.813 (2.13)	1.07 (1.5)	1.39 (1.99)
TG 46:1∗	2.20 (1.39)	3.01 (2.23)	2.22 (1.81)
TG 46:2	1.04 (1.49)	1.31 (1.37)	1.36 (1.83)
TG 48:1	4.02 (2.49)	3.00 (1.87)	3.28 (1.92)
TG 48:2∗	2.33 (1.4)	3.48 (2.25)	2.45 (1.80)
TG 48:3	1.04 (7.07)	3.28 (5.22)	2.13 (10.4)
TG 50:1∗	10.2 (5.44)	8.05 (4.13)	8.92 (4.94)
TG 50:2	8.17 (4.32)	7.23 (3.96)	7.68 (3.48)
TG 50:3	2.09 (1.07)	2.00 (1.13)	1.95 (0.915)
TG 51:2∗	1.12 (0.679)	0.170 (0.965)	0.994 (0.936)
TG 52:2	25.8 (11.9)	28.8 (11.3)	26.1 (11.4)
TG 52:3	12.4 (4.65)	13.5 (6.14)	12.0 (4.93)
TG 52:4	2.63 (1.29)	2.83 (1.53)	2.46 (1.14)
TG 54:3∗	6.84 (3.35)	10.8 (5.01)	7.42 (5.05)
TG 54:4∗	3.07 (1.67)	4.80 (2.62)	3.20 (2.29)
TG 54:5∗	1.08 (0.606)	1.44 (0.821)	1.13 (0.598)
TG 54:6	0.637 (0.806)	0.39 (0.668)	0.464 (0.904)

*PE*, phosphoethanolamine.

Each fatty acid number corresponds to the number of carbon atoms: number of double bonds in the fatty acid.

∗ *P* < .0006 (between breast-fed and formula-fed infants).

**Table III:** Lipid differences between paired samples at 3 months and 12 months of age (N = 45) (median [IQR]) per thousand (‰)

	3-mo, median (IQR), N = 45	12-mo, median (IQR), N = 45
Total cholesterol	154 (50.7)	169 (29.4)
CEs		
CE 16:0	5.80 (3.56)	6.04 (1.07)
CE 16:1	0.684 (2.37)	1.80 (0.965)
CE 18:1	16.6 (7.03)	15.9 (3.49)
CE 18:2∗	19.6 (7.14)	28.8 (9.71)
CE 20:4	1.39 (1.09)	1.82 (1.22)
Phospholipids		
PCs		
PC 32:0∗	9.64 (3.13)	7.48 (1.92)
PC 34:3	6.22 (2.26)	6.14 (1.41)
PC 34:1	76.2 (21.4)	75.5 (14.4)
PC 34:2∗	98.5 (31.7)	130 (21.4)
PC 35:2	2.60 (1.13)	2.81 (0.96)
PC 36:1	15.2 (4.17)	11.6 (5.01)
PC 36:2	72.0 (14.7)	66.6 (10.4)
PC 36:4	35.3 (7.45)	34.6 (9.35)
PC 38:3∗	6.70 (2.98)	4.51 (1.59)
PC 38:4∗	19.5 (4.16)	16.3 (5.53)
PC 38:6∗	12.0 (2.61)	16.2 (8.16)
Lyso-PC 16:0∗	21.5 (5.89)	16.7 (4.95)
Lyso-PC 16:1	0.182 (1.71)	0.756 (0.637)
Lyso-PC 17:0	0.163 (0.917)	0.557 (0.303)
Lyso-PC 18:0∗	10.3 (3.19)	7.16 (2.23)
Lyso-PC 18:1∗	7.02 (2.82)	4.24 (1.4)
Lyso-PC 18:2∗	10.6 (4.27)	6.45 (2.57)
Lyso-PC 20:3∗	1.37 (0.636)	15.6 (12.8)
Lyso-PC 20:4∗	1.75 (0.616)	1.24 (0.406)
Lyso-PC 22:6	1.01 (1.40)	0.714 (0.257)
Lyso-PC-O 16:0	0.297 (1.07)	0.502 (0.142)
PC-O 32:1 and PC-P 32:0	3.02 (5.88)	3.40 (2.22)
PC-O 34:1 and PC-P 34:0∗	3.43 (1.27)	1.46 (0.298)
PC-O 34:3 and PC-P 34:2∗	0.606 (0.521)	1.10 (0.256)
PC-O 38:5 and PC-P 38:4	2.18 (0.593)	2.57 (0.71)
PC-P 34:1 and PC-O 34:2	1.29 (0.499)	1.3 (0.402)
PC-P 35:5	1.09 (1.62)	0.694 (0.334)
PC-P 36:1 and PC-O 36:2	0.605 (1.69)	1.64 (0.812)
PC-P 36:3 and PC-O 36:4∗	2.03 (0.646)	2.38 (0.997)
PC-P 36:4	1.64 (0.417)	1.84 (0.809)
PC-P 38:3 and PC-O 38:4	1.20 (0.800)	1.25 (0.37)
PEs		
PE 36:3	1.04 (1.20)	0.684 (0.163)
PE 36:4	1.67 (1.94)	0.984 (0.386)
PE 40:2	2.47 (7.26)	3.86 (1.38)
Mixed PCs and PEs		
PC 33:1 and PE 36:1∗	3.48 (3.72)	1.12 (0.306)
PC 33:2 and PE 36:2	1.11 (0.68)	1.20 (0.47)
PC 35:1 and PE 38:1	4.23 (3.05)	2.05 (2.04)
PC 35:3 and PE 38:3	0.192 (1.32)	1.07 (0.848)
PC 35:4 and PE 38:4	1.11 (0.681)	1.07 (0.203)
PE 40:5 and PC 37:5	0.39 (1.96)	1.25 (0.548)
SMs		
SM 33:1	2.17 (1.37)	1.64 (0.686)
SM 34:1	48.4 (24.9)	42.9 (13.5)
SM 34:2	4.10 (1.50)	3.95 (1.17)
SM 34:4	2.51 (2.00)	1.79 (1.78)
SM 36:0	1.36 (0.779)	0.892 (0.184)
SM 36:1∗	10.0 (3.95)	7.64 (2.61)
SM 36:2	0.00 (4.88)	1.82 (0.662)
SM 37:1	1.55 (1.24)	0.751 (0.591)
SM 38:1∗	7.73 (2.56)	5.04 (0.992)
SM 39:1∗	2.27 (1.18)	1.48 (0.549)
SM 40:1	11.6 (3.76)	10.8 (2.83)
SM 40:2	6.94 (2.52)	5.64 (1.54)
SM 40:3	0.824 (8.00)	5.52 (2.49)
SM 41:1∗	3.32 (0.993)	4.34 (1.20)
SM 41:2	2.83 (1.63)	2.07 (0.611)
SM 42:1	8.84 (4.59)	9.77 (3.67)
SM 42:2	24.4 (8.90)	22.8 (5.85)
SM 42:3	7.27 (10.5)	5.93 (1.67)
SM 42:4	2.27 (1.15)	2.00 (0.864)
TGs		
TG 44:0	1.38 (2.28)	1.02 (0.457)
TG 44:1	1.32 (1.99)	0.928 (0.836)
TG 46:1	2.19 (2.51)	1.68 (1.94)
TG 46:2	1.24 (1.58)	0.879 (0.619)
TG 48:1	3.81 (2.14)	5.29 (3.97)
TG 48:2	2.31 (2.27)	2.85 (2.33)
TG 48:3	2.13 (8.55)	1.79 (1.35)
TG 49:1	0.317 (1.66)	1.07 (0.739)
TG 50:1	9.44 (4.42)	13.3 (8.4)
TG 50:2∗	8.16 (4.05)	11.6 (6.95)
TG 50:3	2.04 (1.21)	2.78 (1.41)
TG 50:4	0.00 (3.37)	1.08 (0.782)
TG 51:1	0.283 (1.59)	0.933 (0.345)
TG 51:2∗	0.673 (1.12)	1.36 (0.681)
TG 52:2	26.9 (11.8)	32.9 (17.0)
TG 52:3∗	12.6 (4.46)	17.3 (8.01)
TG 52:4∗	2.84 (1.31)	4.17 (2.91)
TG 52:5∗	0.00 (0.969)	0.856 (0.200)
TG 53:2	0.684 (2.83)	1.63 (0.953)
TG 54:3	7.57 (6.52)	7.45 (5.33)
TG 54:4	3.49 (2.48)	4.11 (3.38)
TG 54:5	1.11 (0.559)	1.80 (1.12)

∗ *P* < .0006.

**Table IV:** Associations between 3-month lipid and infancy weight

Lipid	3-mo weight SDS		12-mo weight SDS		3- to 12-mo weight gain SDS	
	Spearman	*P* value	Spearman	*P* value	Spearman	*P* value
PC-O 34:1∗	0.2	.003	0.2	.009	−0.03	.7
PC 34:1∗	0.2	<.0005	0.2	.003	0.01	.9
PC 38:4†	−0.2	<.0005	−0.2	.002	−0.04	.6
SM 34:2†	0.06	.4	−0.07	.3	−0.2	.005
PC-O 36:4†	−0.1	.1	−0.2	.02	−0.2	.004

∗ Lower values in exclusively breast-fed.

† Higher values in exclusively breast-fed.
