# Corrections

**DOI:** 10.1016/j.jpeds.2016.07.010

**Published:** 2016-10

In the article, “Lipidomic Analyses, Breast- and Formula-Feeding, and Growth in Infants,” by Prentice et al (*J Pediatr* 2015;166:276-81), the authors mistakenly omitted the following acknowledgment: *We thank Ieuan Hughes, MD, who established the Cambridge Baby Growth Study cohort and provided the significant intellectual foundation for this project*.

